# Auditory Neural Plasticity in Tinnitus Mechanisms and Management

**DOI:** 10.1155/2020/7438461

**Published:** 2020-07-01

**Authors:** Kunkun Wang, Dongmei Tang, Jiaoyao Ma, Shan Sun

**Affiliations:** ^1^ENT institute and Otorhinolaryngology Department of Eye & ENT Hospital, State Key Laboratory of Medical Neurobiology and MOE Frontiers Center for Brain Science, Fudan University, Shanghai 200031, China; ^2^NHC Key Laboratory of Hearing Medicine, Fudan University, Shanghai 200031, China

## Abstract

Tinnitus, which is the perception of sound in the absence of a corresponding external acoustic stimulus, including change of hearing and neural plasticity, has become an increasingly important ailment affecting the daily life of a considerable proportion of the population and causing significant burdens for both the affected individuals and society as a whole. Here, we briefly review the epidemiology and classification of tinnitus, and the currently available treatments are discussed in terms of the available evidence for their mechanisms and efficacy. The conclusion drawn from the available evidence is that there is no specific medication for tinnitus treatment at present, and tinnitus management might provide better solutions. Therapeutic interventions for tinnitus should be based on a comprehensive understanding of the etiology and features of individual cases of tinnitus, and more high quality and large-scale research studies are urgently needed to develop more efficacious medications.

## 1. Introduction

Tinnitus refers to the perception of sound in the absence of a corresponding external stimulus, and tinnitus is one of the most common complaints of the auditory system globally. A study reviewed all available studies exploring adult populations over a 35-year period and reported a global prevalence of tinnitus ranging from 5.1% to 42.7% and showed that the prevalence increased with aging and noise exposure [1]. Similar epidemiological data regarding adults and younger populations have also been published [2–4]. The intensity of tinnitus varies from subtle noises just above the hearing threshold to high-intensity sounds that cannot be masked by any external sound. Additionally, tinnitus patients almost always suffer from comorbidities that can negatively influence normal life; thus, prevalence estimates for tinnitus provide only a snapshot of the potential population burden of this condition. The percentages of persons having tinnitus accompanied by anxiety and depression are 25.1% and 25.6%, respectively, and these rates greatly exceed those in populations without tinnitus [5, 6]. Concomitant issues with tinnitus can be mild, such as having difficulty concentrating, or can have huge impacts such as recurrent insomnia and severe depression [7, 8]. Because tinnitus can only be heard by the patients in most cases, the adverse effects on daily life can be strong but easily misunderstood by other people, resulting in deterioration of the patient's mental state and even physical condition, and consequently both the quality of life and social function of patients are seriously affected. Because of its associated conditions, tinnitus causes a financial burden on society; for example, the National Health Service of the UK spends about £750 million annually on treating tinnitus patients, at an average cost of £717 per patient [9]. A thorough understanding of tinnitus is essential if its burden on society is to be reduced, and more effective clinical interventions are needed to reduce the adverse impacts on patients' daily life and eventually to eliminate tinnitus completely.

## 2. Tinnitus Classifications and Diagnosis

Tinnitus is divided into many different subtypes, and the most common classification categorizes tinnitus as objective or subjective according to whether or not the sound can be heard by others or measured by instruments. The sounds perceived by tinnitus patients could be tonal ringing, buzzing, clicking, hissing, and other noises. The sounds generally appear in the ear, and sometimes specifically in the head, in which case the condition is referred to as tinnitus cerebri. Objective tinnitus, in which the sound often results from vascular abnormalities and myoclonus, is less common than subjective tinnitus [10, 11].

Accordingly, the diagnosis of tinnitus consists of a subjective evaluation and an objective examination. Questionnaires are generally used for subjective evaluations, and among them, the most frequently used are the Tinnitus Questionnaire, the Tinnitus Handicap Inventory (THI), and the Tinnitus Functional Index (TFI) [12]. In addition to these, there are also some scales used to evaluate the concomitant symptoms of tinnitus that can increase the comprehensive understanding of the patient's situation. There are also psychoacoustic measurements, i.e., tinnitus matching, mainly including tinnitus pitch, loudness, masking level, and residual inhibition, of which pitch matching is the primary sound therapy proven to be effective [13]. Traditional measurements are performed by an audiologist using pure tones, and new approaches involve computer-based or smartphone-based self-administered matching procedures [14].

In terms of objective measurements, palpation and auscultation of the head, neck, and ears are routine tests for objective tinnitus, while in subjective tinnitus electrophysiological measurements are most often used. As the name suggests, otoacoustic emissions refer to the phenomenon in which audio energy is generated within the cochlea and transmitted to the external auditory canal through the middle ear and then released in the form of air vibrations. Otoacoustic emissions represent the functional state of the cochlea and especially the functional state of the outer hair cells (OHCs) [15]. Spontaneous otoacoustic emissions and distortion-product otoacoustic emissions are most commonly used to diagnose tinnitus. Electric response audiometry includes other important electrophysiological measurements, including auditory brainstem response and auditory steady-state evoked response [16]. The function of each part of the auditory pathway can be measured by detecting various biological potentials generated along the auditory pathway [17]. In terms of radiological measurements, functional magnetic resonance imaging (fMRI) has expanded to a broader array of conditions, including tinnitus. Functional changes in the auditory center can be detected by measuring changes in hemodynamics caused by the activity of neurons in response to exogenous acoustic stimulation [18].

## 3. Tinnitus Etiology and Mechanisms

There are many causes of tinnitus, and the mechanisms are very complicated. Secondary tinnitus has obvious causes, such as auditory system diseases like hearing loss and Meniere's disease or systemic diseases such as cardiovascular diseases and diabetes [19, 20]. In contrast, primary tinnitus, or idiopathic tinnitus, is usually of unclear etiology and might not even be associated with sensorineural hearing loss until the time of the clinical visit. In most cases, multiple factors lie behind the etiology of tinnitus.

The pathology leading to tinnitus can be located anywhere from the ear canal to the auditory cortex. Tinnitus often occurs along with hearing loss resulting from noise and aging, and thus, cochlear deafferentation is thought to be a trigger of tinnitus while subsequent changes in the central nervous system are thought to be responsible for the maintenance of tinnitus. This process is also known as neural plasticity [21, 22]. Different neural models within the structures of the central auditory system have been established to interpret the possible mechanisms of tinnitus according to neuroimaging techniques such as electroencephalography (EEG) and fMRI. Hearing impairment leads to deafferentation, which is thought to increase the output of pyramid cells in the dorsal cochlear nucleus according to the hyperactivity model [22]. The inferior colliculus then receives the projection fibers from the cochlear nucleus and shows an increased firing rate, and neurons in the upper structures such as the medial geniculate body of the thalamus and auditory cortex also engage in neuronal hypersynchrony [23–25]. In such cases, the tinnitus is most often related to noise trauma and hearing impairment induced by previous use of chemotherapy drugs [26–28].

Increases in oscillatory activity in the auditory cortex and thalamus have been observed [29, 30], especially in the gamma band oscillatory of the auditory cortex [31, 32], and highly active areas have also been found by fMRI [33, 34]. It has been suggested that tinnitus perception is based on low-frequency oscillations that cause a lateral inhibition imbalance between the normal auditory area and the area with pathological low-frequency activities, thus resulting in high-frequency gamma oscillations [35]. The increases in low-frequency delta band oscillations seen in tinnitus have been replicated in the laboratory along with the reduction in alpha band oscillations [36, 37].

In addition to the above connections within the auditory pathway, a network structure is formed between auditory and nonauditory structures, and this can account for the perception, emotional response, or other reactions to tinnitus. For example, the somatosensory pathway can activate the pyramid cells of the dorsal cochlear nucleus and increase their output [38, 39]. Also, the fibers projecting from the medial geniculate body to the amygdala might facilitate emotional responses to tinnitus. This is because the lateral amygdala receives inputs from neurons in the medial geniculate body and the auditory cortex, and the basal amygdala projects into the hypothalamus in return, thus forming the amygdala auditory feedback loop [40]. EEG measurements have demonstrated enhanced oscillatory electrical activity in the amygdala of tinnitus patients, and the amygdala has been shown by fMRI to respond more strongly to acoustic stimulation in blind individuals for whom the acoustic environment is more important [41, 42].

## 4. Tinnitus Management and Medications

A number of treatments for tinnitus have been developed, and these treatments have shown some efficacy against tinnitus, but there are still a large number of patients around the world who do not have access to effective treatments and who can only try to cope with their symptoms and who must struggle to coexist with them. Therefore, tinnitus is still a clinically unsolved problem, and there is a long way to go to eliminate tinnitus. Treatments are divided into management and medications. Management includes physical therapy, acoustic therapy and psychotherapy, and it is different from the passive reception of medications by patients. In this situation, patients can choose their preferred treatments, and the initiative of involving the patient plays an important role in treating tinnitus. Tinnitus patients and medical professionals are eager to obtain effective drugs that can reduce clinical symptoms, and thus the clinical requirements for such drugs are quite extensive. The use of efficacious drugs to reduce tinnitus might prevent patients from suffering from comorbidities such as depression and anxiety, and thus, even a small effect on tinnitus might have a significant effect on the quality of life.

### 4.1. Tinnitus Management

#### 4.1.1. Psychological Management

The common psychotherapies consist of counseling and cognitive behavioral therapy (CBT). In brief, counseling involves advice and relevant information to facilitate the habituation and comprehension of tinnitus and to help the patient cope with comorbidities brought on by tinnitus such as insomnia and anxiety. Furthermore, counseling is often given in conjunction with other procedures because it is important to help ensure good compliance with other treatments. CBT seeks to rectify and change the patient's maladaptation of cognitive patterns, thus replacing irrational beliefs with reasonable beliefs as a way to eliminate emotional and behavioral problems. CBT contains many elements such as relaxation training and psychological education. In the largest randomized clinical trial involving CBT in tinnitus patients, significant improvement was demonstrated in the quality of life compared with treatment as usual [43]. CBT has been combined with Internet applications in several recent studies, and it was found that both the short-term efficacy of 2 months of treatment and the long-term efficacy of 1 year of treatment were stable and that the quality of life of tinnitus patients was significantly improved [44, 45]. The latest European guidelines also recommend CBT for chronic tinnitus [12], and a Cochrane review also provides evidence that CBT can effectively reduce the impact of tinnitus on a patient's quality of life with few side effects [46]. Thus, it can be concluded that CBT is effective and safe in the treatment of tinnitus.

#### 4.1.2. Physical Therapy

Physical therapy in the treatment of tinnitus involves electromagnetic stimulation, of which repetitive transcranial magnetic stimulation (rTMS) and transcranial direct current stimulation (tDCS) are the most frequently used. tDCS changes the excitability of cortical neurons through the current generated by the depolarization of two scalp electrodes with opposite polarity. rTMS was developed from tDCS and generates pulsed magnetic fields acting on the central nervous system, subsequently changing the membrane potential of cortical neurons and producing induced currents and local electric fields and thus modulating the excitability of the neurons [47]. A Cochrane analysis concluded that the efficacy of short-term rTMS was detectable, but more evidence is needed before conclusions about the long-lasting effect can be drawn [48]. However, it has been shown that rTMS [49, 50] has no significant efficacy on tinnitus patients. Considering the dated references of the Cochrane review mentioned above, it might be more inclined to the invalidity of rTMS. tDCS has shown efficacy measured by TFI [51], THI, and VAS [52]. A review summarized the efficacy of tDCS treatment alleviating tinnitus-related symptoms and suggested further high-quality trials with large sample sizes to determine the efficacy of tDCS on tinnitus as well as any potential side effects [53].

#### 4.1.3. Sound Therapy

Various methods of sound therapy have been developed and have been gaining popularity. The earliest proposed sound therapy was masking, which sought to suppress tinnitus or relieve tinnitus symptoms by listening to a specific external sound matching the loudness of the tinnitus tone under clinical guidance. Despite the wide application of masking therapy, evidence for its efficacy based on controlled studies is still insufficient [54]. Tinnitus retraining therapy (TRT), which aims for tinnitus habituation, was first proposed by Jastreboff and Hazell in 1993 [55]. Strictly speaking, this technique does not belong to pure sound therapy because it consists of a combination of sound therapy and counseling. The sound therapy used in TRT is distinct from that used in masking. In masking therapy, the tinnitus disappears when the masking sound is applied above the loudness of the tinnitus. In contrast, in TRT, the tinnitus and background sound can be perceived at the same time because the tinnitus can only be adapted under conditions in which it is still perceived. Different analogue sounds of natural sounds are applied in TRT: waves, creeks, wind, birdsongs, etc. A Cochrane review [56] reported that it could not reach a conclusion as to whether TRT was effective or not because the treatment principles and methods in most studies included did not strictly correspond to the initial method that Jastreboff and Hazell [55] proposed. Two randomized trials showed the contradictory results between TRT and standard nursing, but it must be noted that most of the subjects in the study showed efficacy of TRT were soldiers, so there was a lack of comparability between the two studies [57, 58]. Thus, further studies on TRT are required.

Music therapy has been incorporated into tinnitus treatment in recent years because individuals' tinnitus profiles often match the frequency spectrum of music. Among a variety of music models, including Heidelberg model music therapy [59] and Neuromonics [60], tailor-made notched music training (TMNMT) is the most prominent. TMNMT modifies enjoyable music by filtering out an octave range of the frequency band centered on the individual's tinnitus frequency, thus reinforcing lateral inhibition and suppressing the overactivity of the neurons in the auditory cortex [61–63]. However, a trial found no improvement after 3 months of training, but a significant reduction in tinnitus loudness was observed in a 1-month follow-up analysis, indicating that the efficacy of TMNMT might need a long time to evolve and stabilize [64]. Two studies have treated tinnitus with TMNMT combined with other methods—tDCS [65] and Ginkgo biloba [66], respectively—and both studies indicated that the subjective perceptions of tinnitus were significantly improved. However, it is not clear where the specific action sites of the three treatment methods are or whether or not there are interactions between them. In addition, these studies lack appropriate designs that can distinguish the effects of two different treatment methods within the same study, and thus, more research is needed to confirm the efficacy of TMNMT alone. A systematic review focused on the research into different treatments online or through smartphones, including TMNMT, CBT, and other methods, and proposed that online treatments could have a positive effect in the daily life of tinnitus patients [67]. Therefore, it can be assumed that with the development of the Internet, treatments of tinnitus through smartphone applications or the Internet might form a new trend in treatment, and such methods might be more convenient and more accepted by patients compared to more traditional forms of treatment.

Hearing aids are also feasible for tinnitus accompanied by hearing loss, and there are several rationales for their use in treating tinnitus. An imbalance exists between excitation and inhibition within the auditory pathway after cochlear injury, and thus, tinnitus might be alleviated by providing extra acoustic input. Hearing aids amplify ambient sounds, which helps patients refocus on other sounds distinct from the tinnitus sound, and thus, hearing aids attenuate the perception of tinnitus [68]. According to the presumed neurological mechanisms of tinnitus, it can be hypothesized that hearing aids decrease afferent inhibition through sound enrichment in the central auditory system and thus achieve the goal of modulating neuroplastic changes. A study compared the effect on tinnitus between conventional hearing aids and frequency-lowering hearing aids in high-frequency hearing loss patients, and both groups found improvement in the quality of life [69]. Another study reported that spectrally notched hearing aids were more effective than unmodified hearing aids in treating tinnitus in hearing loss patients [70]. However, a Cochrane review concluded that there is no evidence to support or refute the application of hearing aids in tinnitus patients with coexisting hearing loss [71]. The hearing aids using notched environment sounds might be more convenient than conventional TMNMT because a specific acoustic environment is not required and patients can benefit from the treatment all the time. However, the studies on hearing aids have only included a small portion of tinnitus patients, and multicenter randomized trials might be helpful in reconfirming the efficacy of hearing aids.

### 4.2. Medications for Tinnitus

As traditional, simple, and acceptable therapeutics, tinnitus medications have huge potential to meet patients' demands, although there is still limited research progress in this area. Rapid improvement in symptoms and the maximization of normal living conditions are eagerly expected for future therapeutics; thus, research into drug treatment still has a long way to go. Among the drugs discussed in this review, the most studied are antidepressants, which are mostly used to solve the problems like depression and anxiety caused by tinnitus. Accompanying the improvement of patients' depression, some patients' subjective perceptions of tinnitus are improved. However, due to the limited comprehension of tinnitus mechanisms, we could not accurately locate the specific targets of antidepressants in tinnitus, so it is hard to judge whether or not antidepressants have other therapeutic effects on tinnitus besides an antidepressant effect. We summarize the tinnitus-related compounds and drugs in Tables 1 and 2.

#### 4.2.1. Psychotropic Drugs

*(1) Benzodiazepines*. Benzodiazepines are increasingly being used to treat nonepileptic diseases, including various mental disorders and pain syndromes, and their application in tinnitus is based on the presumption that tinnitus is affiliated with neuronal hyperactivity in the central auditory system. As positive allosteric modulators of the GABAA receptor [72], benzodiazepines may also reduce the comorbidities of tinnitus such as insomnia and anxiety due to their anxiolytic and hypnotic effects but not their anxiolytic and hypnotic effects. Studies on benzodiazepines in the treatment of tinnitus have included alprazolam and clonazepam. A trial involving 30 patients showed improvement in tinnitus loudness and annoyance as measured on a visual analogue scale (VAS) [73]. In another study, 3 weeks of clonazepam treatment showed obvious improvement in tinnitus loudness, VAS measurements of annoyance, and THI scores [74]. A retrospective study showed that clonazepam doses varying from 0.5 to 1 mg/day with 2–6-month treatment times alleviated tinnitus symptoms in 32% of over 3,000 subjects with vestibular or cochlear disorders [75]. As for alprazolam, two well-designed trials were performed, with the first reporting a reduction of tinnitus loudness in 76% of patients when measured by a tinnitus synthesizer compared to 5% of patients when measured by VAS [76]. Interestingly, the second trial showed improvement in VAS score but not in tinnitus loudness or THI score [77]. These studies suggested the potential of benzodiazepines in tinnitus treatment, but in consideration of the inconsistent performances in different scales and the adverse effect profiles, there is no mature evidence to recommend benzodiazepines as a pharmacologic approach to tinnitus. However, it is worth further exploring the refined mechanism of action in order to obtain better clinical results, at least improvement of the adverse symptoms brought about by tinnitus, and thus, more studies should be carried out to better understand the potential efficacy of benzodiazepines.

*(2) Dopaminergic and Antidopaminergic Drugs*. Dopaminergic pathways are speculated to be related to tinnitus in various areas of the brain, and it has been observed that dopamine is present in the first synaptic complex of the auditory pathway between IHCs and neurons, thus enabling the study of dopaminergic and antidopaminergic drugs on tinnitus [78, 79]. A study of the dopamine agonist pramipexole showed alleviation of presbycusis-related tinnitus [80]. the dopamine antagonist sulpiride was also found to be effective in reducing tinnitus perception both alone and in combination with either melatonin or hydroxyzine [81, 82]. Nevertheless, negative results for the dopamine agonist piribedil were also reported, and 19 out of 75 participants quit the study owing to side effects such as nausea and dizziness [83]. This demonstrates our insufficient understanding of the mechanisms involved in dopaminergic-related tinnitus, and thus, further studies are required to determine whether or not dopamine agonists or antagonists can benefit tinnitus sufferers despite their potential side effects.

**Figure 1:**
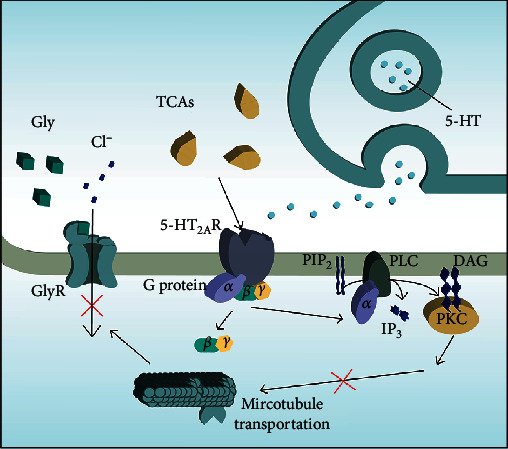
Possible therapeutic mechanism of TCAs on tinnitus. TCAs block the 5-HT_2A_ receptor, thus preventing 5-HT from binding to the 5-HT_2A_ receptor and activating the PLC-IP_3_/DAG-PKC pathway and thus impairing downstream microtubule-dependent glycine receptor transport. This in turn acts as a barrier to glycine receptor binding on the surface of the cell membrane followed by a reduction of chloride influx and thus leading to a decrease in tonic current and an increase in intracellular potential and excitability. TCAs: tricyclic antidepressants; 5-HT_2A_R:5-hydroxytryptamine receptor _2A_ receptor; PIP_2_: phosphatidylinositol 4,5-bisphosphate; PLC: phospholipase C; DAG: diacylglycerol; PKC: protein kinase C; IP_3_: inositol 1,4,5-trisphosphate.

*(3) Antidepressants*. Tinnitus is almost always accompanied by various psychological symptoms, of which depression and anxiety are the most commonly reported. In the auditory cortical neurons, it was found that the 5-hydroxytryptamine_2A/C_ (5-HT_2A/C_) receptor agonist 2,5-dimethoxy-4-iodoamphetamine (DOI) could suppress the glycine receptor-mediated tonic current. DOI did not compete with glycine; instead, it initially activated the 5-HT_2A/C_ receptor, which impaired microtubule-dependent glycine receptor transport resulting in a decrease in tonic current [84]. Seeing that tinnitus is associated with decreased lateral inhibition of the cortex, 5-HT receptor agonists might further reduce the glycine receptor-mediated tonic current through lateral inhibition of hearing; thus, there are reasons to believe that the 5-HT receptor participates in the pathogenesis of tinnitus. Interestingly, tricyclic antidepressants (TCAs) and selective serotonin reuptake inhibitors (SSRIs), which are the most commonly used antidepressants in the treatment of tinnitus, both inhibit serotonin reuptake, thus increasing the release of serotonin by elevating the endogenous serotonin concentration in the synaptic membrane followed by downregulation of 5-HT_1A_ receptors in the presynaptic membrane. This seems to be paradoxical, but the fact that TCAs also have a role in blocking 5-HT_2A_ shows that diverse pathways may contribute to the efficacy of antidepressants [85] (Figure 1). In fact, little is known of the pathogenesis of tinnitus, especially with regard to explaining the efficacy of antidepressant treatments. It should be noted that the ideal drugs for treating tinnitus should both reduce the awareness of tinnitus and improve the patient's emotional status, thus increasing the quality of life of patients.

In two studies looking into the TCA nortriptyline [86, 87], patients with severe tinnitus showed improvement in depression and tinnitus loudness after treatment, and a greater reduction in symptoms was observed in subjects suffering from more severe depression; in other words, nortriptyline appears to be especially suitable for severely depressed tinnitus patients. Podoshin et al. [88] found no significant advantage of another TCA, amitriptyline, but a later study showed a distinct decrease in tinnitus severity in 95% of the experimental group [89]. SSRIs have also been applied in cases of tinnitus. Patients with depression and high risk of disabling tinnitus showed significant improvement after sertraline treatment, but it is important to note the relatively high dropout rate of 17% after sertraline administration [90]. Paroxetine treatment exhibited little difference in tinnitus loudness or other psychological measures except for tinnitus aggravation [91]. In addition, it is noteworthy that there have been cases where tinnitus was induced or worsened during or after antidepressant treatment [92–94].

There is also a report of applying the compound Deanxit along with clonazepam in tinnitus patients, and the results indicated that Deanxit was more effective than placebo whether used alone or combined with clonazepam [95]. Trazodone is considered an atypical antidepressant and is classified as a serotonin antagonist and a reuptake inhibitor with complex pharmacological effects including both agonistic and antagonistic effects on the serotonin system [96]. Thus, dual mechanism might increase serotonin levels in the central auditory pathway, but a study found no difference between trazodone treatment and placebo [97]. Tianeptine belongs to the TCAs in terms of its structure, but its unique pharmacological effect of increasing 5-HT reuptake in the presynaptic membrane is different from the traditional TCAs. In a study of tianeptine treatment, the control group was made up of tinnitus patients without depressive mood, and tinnitus as measured by THI and depression as measured by the Beck Depression Inventory were significantly decreased after tianeptine treatment [98]. Therefore, whether the effect of antidepressants on 5-HT can restore tonic inhibition or reduce central excitability deserves further exploration. However, in view of the side effects of both SSRIs and TCAs, a more rigorous clinical scheme is needed to produce convincing results.

#### 4.2.2. Ion Channel Drugs

**Figure 2:**
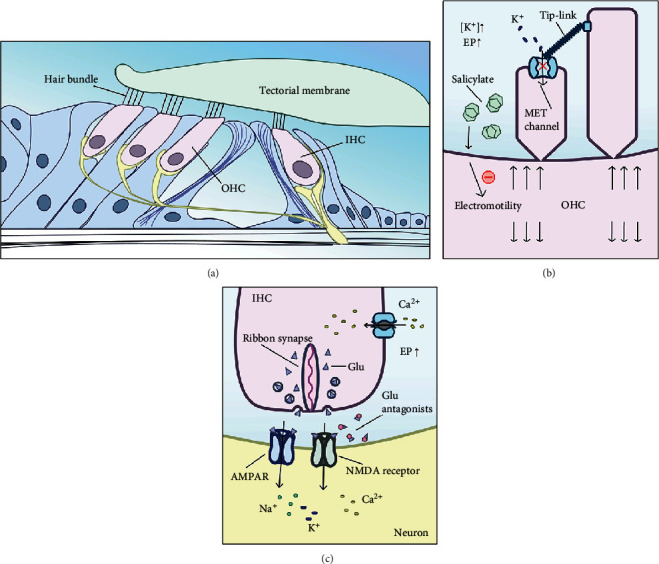
Possible therapeutic mechanism of glutamate antagonists on salicylate-induced tinnitus. (a) Schematic diagram of the organ of Corti. (b) Salicylate can inhibit the electromotility of OHCs, which reduces the opening probability of MET channels, downregulates the influx of K^+^ through the MET channels, and increases the EP. The sets of three down and three up arrows represent the longitudinal extension of electromotility in OHCs. (c) The increased EP is followed by opening of the voltage-gated Ca^2+^ channels, fusion of the synaptic ribbon to the cytomembrane of the IHCs, and release of glutamate, and thus cochlear fibers depolarize abnormally and tinnitus occurs. Glutamate antagonists can inhibit the process by blocking AMPARs and NMDA receptors. IHC: inner hair cell; OHC: outer hair cell; EP: endocochlear potential; MET: mechanoelectrical transduction; NMDA receptor: N-methyl-D-aspartic acid receptor; AMPAR: *α*-amino-3-hydroxy-5-methyl-4-isoxazole-propionicacid receptor.

*(1) Glutamate Receptor Antagonists*. Glutamate receptor antagonists reported in the treatment of tinnitus are all aimed at ion channel receptor subtypes. Previous studies have suggested that regulation of the endocochlear potential (EP) by OHCs contributes to increased spontaneous cochlear activity. A portion of the mechanoelectric transduction (MET) channels are open at rest in inner hair cells (IHCs), and alterations of the EP can lead to corresponding electrical activities in IHCs including depolarization and hyperpolarization [21]. By limiting or preventing the current through their MET channels, OHCs can reduce the opening probability of MET channels. Downregulating the influx of K^+^ through MET channels increases the EP, which leads directly to depolarization, the opening of voltage-gated Ca^2+^ channels, and fusion of the synaptic ribbon to the cytomembrane of IHCs. The release of glutamate at ribbon-type synapses is the result of IHC depolarization and causes cochlear fibers to depolarize [99, 100]. This might play a pivotal role by amplifying N-methyl-D-aspartic acid- (NMDA-) mediated neurotransmission in tinnitus, and the increased cochlear firing rate caused by glutamate has been found in salicylate-induced tinnitus through the activation of NMDA receptors [101, 102], which are expressed at all synapses of the lower auditory pathway in mammals [103]. Salicylate can inhibit the activity of cyclooxygenase and increase the concentration of arachidonic acid in the cytomembrane of cochlear nerve fibers, thus increasing the possibility of NMDA receptor opening by altering the mechanical properties of the cytomembrane and thus bringing about both peripheral and central effects [104]. In addition to this, salicylate is also reported to reduce the electromotility of OHCs, which may have an effect on the mediation of stereocilium bundle deflection that influences the probability of MET channel opening [105, 106] (Figure 2).

Research using memantine has indicated a significant reduction of symptoms in tinnitus triggered by both salicylate [107, 108] and noise trauma in rats [109]. It was also seen that memantine attenuated the increased level of NMDA receptor subunit 2B protein, which was markedly induced by salicylate and might be associated with inhibition of NMDA receptors in the auditory cortex [108]. Washout of the memantine was performed after clear efficacy was observed, and tinnitus-related behavioral manifestations were partially reduced, which might be interpreted as either a prolonged effect of memantine or an insufficient washout period that failed to entirely eliminate the memantine [109]. A study observed a lack of suppression of tinnitus at lower memantine doses, which the investigators concluded to be the limitation of the conditioned suppression technique [110]. Another trial showed less satisfactory results due to poor improvement in the THI score and relatively serious side effects. In addition, the appearance of a significant interaction between efficacy and treatment order indicated the possibility that a delayed carryover effect exists for treatment with memantine along with a more pronounced placebo effect in the group previously treated with memantine [111]. The low affinity and rapid off-rate of memantine give it a more tolerable and lower adverse event profile despite having similar activity on the NMDA receptor as MK-801, another NMDA receptor antagonist [112].

A study reported that after salicylate injection, the levels of glutamate and ascorbate in the auditory cortex increased significantly and that MK-801 attenuated these reactions, suggesting that MK-801 might act as a neuroprotective agent against hyperactivity in salicylate-induced tinnitus [113]. In addition, systemic distribution of MK-801 might also decrease the hyperactivity of the lower auditory pathway, for instance, in the dorsal cochlear nucleus [114]. This indicates that MK-801 might play a role mainly in the central auditory structures. Tinnitus patients treated with neramexane had a consistent decrease in THI scores in the higher-dose groups after 8 weeks of initial treatment [115]. Investigators also explored the efficacy of acamprosate, which showed a beneficial effect after at least 60 days of treatment, and approximately 87% of the patients taking acamprosate showed an improvement [116]. The efficacy of acamprosate was confirmed in another trial by showing a higher alleviation rate of 92.5% [117]. These studies suggest that NMDA receptor antagonists such as memantine, MK-801, and acamprosate may be worthy of further exploration for the treatment of tinnitus. However, Auris Medical announced that the phase III clinical trial of AM-101, an NMDA receptor antagonist, failed to achieve the primary endpoint in acute tinnitus, despite positive indications published during early phases of the trial [118]. In addition, another glutamate receptor, *α*-amino-3-hydroxy-5-methyl-4-isoxazole-propionicacid receptor (AMPAR), might also be involved in the pathogenesis of salicylate-induced tinnitus through increased expression on the excitatory neurons of the auditory cortex [119], for which BGG492 has been shown to reduce VAS values for chronic subjective tinnitus loudness and annoyance in a phase II study.

Numerous studies have looked at drugs targeting the ion channels of cells, but the results so far have been unsatisfactory. With progress in research on the mechanisms behind tinnitus in the central nervous system, our understanding of cell function such as neuron activity has gradually increased, but there have been no large leaps in our understanding of the molecular mechanisms behind tinnitus. Perhaps a better understanding of the mechanisms and heterogeneity of tinnitus will provide new sites for drug activity in the future.

*(2) Other Channel Modulators*. Generally, voltage-gated potassium channels are the predominating determinants of the intrinsic excitability of cells. Among them, Kv3.1 is a high-threshold potassium channel that is expressed in fast-spike neuron plasmalemma within the central auditory system [120]. The high voltage-activated potassium current, which might be the cause of the spontaneous hyperactivity of characteristic neurons in high-frequency burst firing, is downregulated by noise exposure in rats [121]. AUT00063 is a newly developed central neuron-targeted drug, and as a potent and selective regulator of Kv3.1 and Kv3.2 voltage-gated channels, AUT00063 can shift neurons' activated voltage dependence to a lower negative potential from which it is more difficult to obtain an elevation of intracellular potential. Indeed, AUT00063 has been shown to inhibit spontaneous hyperactivity induced by noise exposure in the fusiform cells of the dorsal cochlear nucleus, as well as multiunit activity recorded in the inferior colliculus in mice [122, 123]. However, a daily dose of 800 mg AUT00063 for 28 days showed safety and tolerance but resulted in no change in TFI scores [124]. Patients with intermittent typewriter-like tinnitus all responded positively to carbamazepine, and therefore, it is speculated that carbamazepine alleviates tinnitus by hindering the recovery from inactivation of the voltage-gated sodium channel and thus suppressing subsequent ephaptic axonal transmission in the cochlear nerve [125].

Gabapentin is a synthesized structural analog of GABA, and it has been found to bind to the *α*_2_-*δ* subunit of voltage-dependent calcium channels with high affinity [126]. There have been attempts at using gabapentin in the treatment of tinnitus, and a controlled trial in patients with tinnitus induced by acoustic trauma showed significant improvement in tinnitus annoyance and loudness [127]. Another pilot study with gabapentin found a significant improvement in tinnitus annoyance and a decrease in tinnitus handicap [128]. However, further trials did not detect any benefit on tinnitus by gabapentin [129, 130], and in another study, beneficial effects were only reported in the subgroup of tinnitus patients with hypertension, diabetes, or dyslipidemia, and there was no difference on the group level [131]. In addition, the use of anticonvulsants including gabapentin in treating tinnitus was analyzed in a Cochrane review, which showed no evidence for a clinical effect and doubtful clinical significance as well as a high rate of side effects (18% of trial participants) in the treatment of tinnitus [132].

#### 4.2.3. Antioxidants

Oxidative stress is involved in the pathogenesis of many diseases, including tinnitus. Several studies showed that oxidants were elevated accompanied by a reduction of antioxidants in tinnitus patients [133–136], and a study reported similar results in the internal jugular and brachial vein blood of acute idiopathic tinnitus patients. However, due to the exceedingly small portion that the inner ear occupies in the total brain effluent, we cannot jump to the conclusion that both endothelial dysfunction and oxidative stress originate from the inner ear microcirculation [137]. A study speculated that impairment of nitric oxide production could result in vascular dysregulation [138]. Oxidative stress damages the labyrinthine neurosensory epithelium and the vestibulocochlear nerve, as well as central auditory pathway, and the neurosensory epithelium is especially at risk of cochlear lesions induced by reactive oxygen species (ROS) [139–142]. One of the most important effects of ROS, lipid peroxidation, might mediate apoptosis of auditory neurons and ciliated cells [143]. In addition, ROS can cause endothelial dysfunction, which is more obvious in the terminal microcirculation. Thus, the established hypothesis is that ROS might cause damage to the stria vascularis and the capillary networks of the planum semilunatum [144–147].

Khan et al. reported that the application of coenzyme Q10 (CoQ10) alone only reduced Tinnitus Questionnaire scores in those who lacked CoQ10 [148]. However, in another CoQ10 study, patients undergoing cisplatin chemotherapy retained stable concentrations of ROS and fewer instances of hearing impairment and tinnitus, but because the efficacy was from a mixture of several different compounds, it is difficult to discriminate the efficacy of CoQ10 alone [149]. CoQ10 mainly acts on mitochondrial and cellular membranes, and it is involved in the electron transfer chain as part of the energy generation process. Therefore, the level of CoQ10 in plasma can only provide limited information about oxidative defense [150, 151]. Zinc is a structural component of superoxide dismutase which is the primary line of defense against oxidative stress. A significant improvement in the THI score was achieved in noise-induced hearing loss-associated tinnitus patients treated by zinc [152], while another study showed an adverse result in elderly tinnitus patients [153]. In any case, a Cochrane review concluded that there is no evidence of efficacy of oral zinc supplementation in adults with tinnitus [154]. The antioxidant N-acetyl-L-cysteine (NAC) was able to alleviate noise-induced hearing loss in soldiers, thus suggesting that NAC could attenuate the toxic effect of acoustic trauma and it might represent a new compound for treating inner ear injuries as well as tinnitus [155].

There are also reports of drugs that can improve the microcirculation of the cochlea by improving the blood flow and thus increasing the clearance of ROS through the bloodstream. Pentoxifylline has vasodilation activity and thereby increases blood flow, and sulodexide has antithrombotic and anticoagulant activities, both of which have been shown to have positive effects in tinnitus patients, mainly by improving the subjective perception and emotional response to tinnitus [156, 157]. However, the changes of inner ear microcirculation in tinnitus are not completely clear yet, and various antioxidants still need to pass through the blood-labyrinth barrier; thus, the therapeutic effect is not very precise.

#### 4.2.4. Herbal Medicines

A retrospective study reported that some medicinal plants, including Asteraceae, Lamiaceae, and Ginkgo biloba, had been used to treat tinnitus in Iran, but there was little concrete evidence for the efficacy and mechanism of these herbal medicines [158]. Ginkgo biloba contains various agents, including ginkgo-flavone glycosides, which can scavenge free radicals, and terpenoids, which act as antagonists of platelet-activating factor [159]. Looking through studies on Ginkgo biloba, we found contrary treatment effects on tinnitus, and a Cochrane review concluded that there was limited evidence to support the effectiveness for patients whose primary complaint was tinnitus [160]. This might be due to a lack of standard usage, a lack of optimal doses, and a lack of standard methodological measurements of efficacy [149, 161–164]. The efficacy of tinnitus is likely to be the mixture of multiple ingredients, which might not be conducive to guiding the treatment and avoiding adverse effects.

#### 4.2.5. Dietary Supplements

In addition to their usage in treating neurological disorders and oxidative stress, dietary supplements have also been applied in the treatment of tinnitus. It is verified that mixed supplements of vitamins and phospholipids could reduce tinnitus intensity and subjective symptoms along with reduced ROS levels [143]. However, later, no benefit was found from the compounds (Ginkgo biloba, *α*-lipoic acid, vitamin C, papaverine hydrochloride, and vitamin E) in elderly patients with tinnitus [164]. Vitamin B12 deficiency is related to axonal degeneration and demyelination followed by the death of neurons. Efficacy was only shown in tinnitus patients who had vitamin B12 deficiency [165–167]. There have been studies investigating the efficacy of melatonin, either as monotherapy or in combination with sulodexide or vitamins. The plasma level of melatonin might be associated with tinnitus and might improve tinnitus-related sleep problems [168–171] and might only show obvious treatment effects in tinnitus patients with insomnia [172, 173]. Melatonin might not be able to directly improve the symptoms of tinnitus at all, but it might solve the sleep problems caused by tinnitus.

### 4.3. Discussion on Tinnitus Medications and Management

In terms of mechanisms, tinnitus management is mainly based on the neurophysiological changes of tinnitus that have been elaborated in different neurophysiological models [174–177], and such management takes a more holistic approach compared with the molecule-mechanism medications for tinnitus. Clinically, tinnitus management is more intuitive based on the current electrophysiological methods for diagnosing tinnitus. With the popularization of smartphone apps and the Internet, tinnitus self-management procedures based on sound therapy or psychological consultation can be more readily put into use, and these are more likely to be promoted clinically due to their safety and ease of use. Currently, the European guidelines only strongly recommend CBT as a treatment for tinnitus. The safety of sound therapy has been verified, but there is no evidence of its efficacy. However, according to the lateral inhibition theory of tinnitus frequency-related cortical neurons, notched sounds can cause neural plasticity by constantly stimulating and changing the excitability of neurons, and thus, sound therapy is still a promising treatment for alleviating the subjective symptoms of tinnitus.

It is worth noting that the studies of the molecular mechanisms of tinnitus have also made some progress [178, 179]. Most of the medications treating tinnitus are off-label use, and drugs specifically developed for tinnitus have been proven ineffective [118, 122, 124]. The efficacy of the same drug observed in different studies might be inconsistent, and the incidence of side effects is higher than tinnitus management. Also, no medication is recommended to treat tinnitus in the guidelines, and in view of the side effects of different medications, they should be applied cautiously. The treatment of tinnitus presents a challenge, and there are several obstacles to the development of effective pharmaceutical treatments. For example, the heterogenetic nature of tinnitus makes it difficult to identify the corresponding characteristics of different tinnitus subtypes. Drugs applied in the treatment of tinnitus, regardless of their off-label usage, might reduce tinnitus-related complications and offer minor alleviation of tinnitus perception, but none are able to offer curative treatment. On the molecular level, neurotransmitters at all levels of the auditory pathway structures can be potential targets for drugs. For example, benzodiazepines are applied to treat tinnitus because of their positive allosteric regulatory effect on central GABA receptors, and it is very interesting that these drugs can both increase GABAergic inhibition and decrease GABAergic inhibition in the medial geniculate body [180]. Also, the nonauditory pathway structures, such as the areas associated with emotion, may be affected by antidepressants, and it is worth studying whether or not the therapeutic effect of TCAs is related to their antagonistic effect on 5-HT receptors in addition to the improvement of tinnitus-related depression.

On the whole, although it seems that there is no clear efficacy for medications targeting specific molecules due to the subjectivity and heterogeneity of tinnitus [181, 182], objective improvement is also difficult to observe in both the management and medical treatment of tinnitus patients. Thus, suggestions given by clinicians are primarily along the lines of “Don't worry about it” or “Don't pay attention to it”; that is to say, subjective adaptation is still the main method for patients in coping with tinnitus.

## 5. Conclusion

At present, standard therapeutics for tinnitus are absent. However, although there is no evidence to support the use of medications for tinnitus, they might still have a place in treatments as we gain a better understanding of the pathogenesis of tinnitus. Our understanding of tinnitus is limited, and this might be overcome by more characteristic animal models and subsequently more high-quality clinical trials. Thus, in the absence of an effective therapeutic protocol for tinnitus, the treatment of tinnitus mainly focuses on eliminating disturbing symptoms in line with different situations. For example, patients suffering from tinnitus and depression can be treated with CBT and drugs, and hearing aids can be considered for hearing loss patients disturbed by tinnitus. In any case, clinicians should respect the individual's opinions and choose the most suitable treatment plan.

## Figures and Tables

**Table 1:** Clinical trials of psychotropic drugs for tinnitus.

Drugs	Authors & year	Effect targets	Conclusions
Gabapentin	[127]	Not clear, enhances the activity of GABA	Loudness reduced by 11–13.3 dB in the acoustic trauma subgroup
	[128]		Not effective
	[130]		Not effective
	[129]		Not effective
	[131]		Not effective
Clonazepam	[73]	Antagonizes GABAR	VAS-A decreased by 2.9
	[74]		Loudness reduced by 4 dB; VAS-L/A and THI decreased by 3/4 and 4, respectively
Alprazolam	[76]	Not clear, interacts with GABAR	Loudness reduced in 76% of the treatment group
	[77]		VAS decreased by 2.83
Sulpiride	[81, 82]	Antagonizes DR_D2_	VAS-P decreased by 1.5
			VAS-P decreased by 1.4
Piribedil	[83]	Agonizes DR_D2/3_	Not effective
Pramipexole	[80]	Activates DR_D3_	Percentage of loudness reduced by 15 dB with statistical significance
Nortriptyline	[86]	SSRI; 5-HT_2A_R blocker	Loudness reduced by 10 dB
	[87]		HAMD and ITI decreased by 3.7 and 0.6, respectively; loudness reduced by 6.4 dB
Amitriptyline	[88]	SSRI; 5-HT_2A_R blocker	No more effective than biofeedback
	[89]		ATAQ decreased by 2.95 and 2.55 in the right and left ear, respectively
Sertraline	[90]	SSRI	TSQ decreased by 4.68; VAS-L decreased by 1.51
Paroxetine	[91]	SSRI	Not effective
Trazodone	[97]	SSRI; 5-HT_2A/C_R blocker	Not effective
Tianeptine	[98]	SSRE	THI decreased by 8.6; BDI decreased by 9.18
Deanxit	[95]	5-HT_2A_R/DR_D1/2_ blocker	VAS-A/S and TQ decreased by 9.5/1.2 and 11.0, respectively

GABAR: gamma aminobutyric acid receptor; VAS-L/A/P/S: visual analog scale of loudness/annoyance/tinnitus perception/somatization; THI: tinnitus handicap inventory;3,4-dhydroxyphenethelamine receptor_D1/2/3_; SSRI/E: selective serotonin reuptake inhibitor/enhancer; 5-HT_2A/C_R: 5-hydroxytryptamine_2A/C_ receptor; HAMD: Hamilton Depression Scale; ITI: multidimensional pain inventory of tinnitus interference; ATAQ: 10-point scale from of the American Tinnitus Association questionnaire; TSQ: Tinnitus Severity Questionnaire; BDI: Beck Depression Inventory; NMDAR: N-methyl-D-aspartic acid receptor; TS: Tinnitus Scale; ETC: electron transport chain; TQ: Tinnitus Questionnaire; SOD: superoxide dismutase; 11-PBS-L/A: 11-point box scale-loudness/annoyance; TSS: Tinnitus Severity Score.

**Table 2:** Clinical trials of other medications for tinnitus.

Drugs	Authors & year	Effect targets	Conclusions
Memantine	[111]	NMDAR antagonist	Not effective
Neramexane	[115]	NMDAR and 5-HT_3_R	Not effective
Acamprosate	[116]	Glu antagonist; GABA agonist	TS decreased by 3.87
	[117]		Higher alleviating rate (92.5% vs. 12.5%)
AM-101	[118]	NMDAR antagonist	Not effective
AUT00063	[124]	Modulates Kv_3.1/3.2_ channels	Not effective
CoQ10	[148]	Component of ETC	TQ scores decreased by 14
	[149]	Antioxidants	Lower tinnitus incidence rate (11.1% vs. 62.5%)
Zinc	[153]	Component of SOD	Not effective
	[152]		THI reduced by 8.3
Pentoxifylline	[157]	Improves inner ear perfusion	VAS-P decreased by 5 and 3 compared to pretreatment and placebo, respectively
Sulodexide	[156]	Antithrombotic/anticoagulant	THI and Mini-TQ reduced by 10.3 and 3.9, respectively
Ginkgo biloba	[162]	Antioxidants	Mini-TQ and 11-PBS-L/A reduced by 2.19 and 0.74/1.06, respectively
	[163]		THI score reduced by >20
	[164]		Not effective
Vitamin B12	[165]	Maintains the function of myelin, etc.	Not effective
	[167]		TSS reduced by 8.2 in patients with B12 deficiency
Melatonin	[172]	Scavenges free radicals, etc.	No significance effect measured by THI
	[171]		THI reduced by 10.4 compared with placebo
	[168]		THI scores decreased by 4.6
	[173]		Higher alleviating rate (57% vs. 25%)

GABAR: gamma aminobutyric acid receptor; VAS-L/A/P/S: visual analog scale of loudness/annoyance/tinnitus perception/somatization; THI: tinnitus handicap inventory;3,4-dhydroxyphenethelamine receptor_D1/2/3_; SSRI/E: selective serotonin reuptake inhibitor/enhancer; 5-HT_2A/C_R: 5-hydroxytryptamine_2A/C_ receptor; HAMD: Hamilton Depression Scale; ITI: multidimensional pain inventory of tinnitus interference; ATAQ: 10-point scale from of the American Tinnitus Association questionnaire; TSQ: Tinnitus Severity Questionnaire; BDI: Beck Depression Inventory; NMDAR: N-methyl-D-aspartic acid receptor; TS: Tinnitus Scale; ETC: electron transport chain; TQ: Tinnitus Questionnaire; SOD: superoxide dismutase; 11-PBS-L/A: 11-point box scale-loudness/annoyance; TSS: Tinnitus Severity Score.

## References

[1] McCormack A., Edmondson-Jones M., Somerset S., Hall D. (2016). A systematic review of the reporting of tinnitus prevalence and severity. *Hearing Research*.

[2] Park B., Choi H. G., Lee H. J. (2014). Analysis of the prevalence of and risk factors for tinnitus in a young population. *Otology & Neurotology*.

[3] Gilles A., van Hal G., de Ridder D., Wouters K., van de Heyning P. (2013). Epidemiology of noise-induced tinnitus and the attitudes and beliefs towards noise and hearing protection in adolescents. *PLoS One*.

[4] Mahboubi H., Oliaei S., Kiumehr S., Dwabe S., Djalilian H. R. (2013). The prevalence and characteristics of tinnitus in the youth population of the United States. *Laryngoscope*.

[5] Shargorodsky J., Curhan G. C., Farwell W. R. (2010). Prevalence and characteristics of tinnitus among US adults. *The American Journal of Medicine*.

[6] Krog N. H., Engdahl B., Tambs K. (2010). The association between tinnitus and mental health in a general population sample: results from the HUNT study. *Journal of Psychosomatic Research*.

[7] Bhatt J. M., Bhattacharyya N., Lin H. W. (2017). Relationships between tinnitus and the prevalence of anxiety and depression. *Laryngoscope*.

[8] Crönlein T., Langguth B., Pregler M., Kreuzer P. M., Wetter T. C., Schecklmann M. (2016). Insomnia in patients with chronic tinnitus: cognitive and emotional distress as moderator variables. *Journal of Psychosomatic Research*.

[9] Stockdale D., McFerran D., Brazier P. (2017). An economic evaluation of the healthcare cost of tinnitus management in the UK. *BMC Health Services Research*.

[10] Elgoyhen A. B., Langguth B., de Ridder D., Vanneste S. (2015). Tinnitus: perspectives from human neuroimaging. *Nature Reviews. Neuroscience*.

[11] Bauer C. A. (2018). Tinnitus. *New England Journal of Medicine*.

[12] Cima R. F. F., Mazurek B., Haider H. (2019). A multidisciplinary European guideline for tinnitus: diagnostics, assessment, and treatment. *HNO*.

[13] Pantev C., Okamoto H., Teismann H. (2012). Music-induced cortical plasticity and lateral inhibition in the human auditory cortex as foundations for tonal tinnitus treatment. *Frontiers in Systems Neuroscience*.

[14] Kim T. S., Yakunina N., Ryu Y. J., Chung I. J., Nam E. C. (2017). Self-administered tinnitus pitch matching versus a conventional audiometric procedure. *Audiology & Neuro-Otology*.

[15] Brownell W. E. (1990). Outer hair cell electromotility and otoacoustic emissions. *Ear and Hearing*.

[16] Engel S., Markewitz R. D. H., Langguth B., Schecklmann M. (2017). Paired associative stimulation of the temporal cortex: effects on the auditory steady-state response. *Frontiers in Psychiatry*.

[17] Jackson R., Vijendren A., Phillips J. (2019). Objective measures of tinnitus: a systematic review. *Otology & Neurotology*.

[18] Leaver A. M., Turesky T. K., Seydell-Greenwald A., Morgan S., Kim H. J., Rauschecker J. P. (2016). Intrinsic network activity in tinnitus investigated using functional MRI. *Human Brain Mapping*.

[19] Bener A., al-Hamaq A. O. A. A., Abdulhadi K., Salahaldin A. H., Gansan L. (2017). Interaction between diabetes mellitus and hypertension on risk of hearing loss in highly endogamous population. *Diabetes and Metabolic Syndrome: Clinical Research and Reviews*.

[20] Gibrin P. C. D., Melo J. J., de Moraes Marchiori L. L. (2013). Prevalence of tinnitus complaints and probable association with hearing loss, diabetes mellitus and hypertension in elderly. *CoDAS*.

[21] Noreña A. J. (2015). Revisiting the cochlear and central mechanisms of tinnitus and therapeutic approaches.. *Audiology and Neurotology*.

[22] Henry J. A., Roberts L. E., Caspary D. M., Theodoroff S. M., Salvi R. J. (2014). Underlying mechanisms of tinnitus: review and clinical implications. *Journal of the American Academy of Audiology*.

[23] Norena A. J., Eggermont J. J. (2003). Changes in spontaneous neural activity immediately after an acoustic trauma: implications for neural correlates of tinnitus. *Hearing Research*.

[24] Kalappa B. I., Brozoski T. J., Turner J. G., Caspary D. M. (2014). Single unit hyperactivity and bursting in the auditory thalamus of awake rats directly correlates with behavioural evidence of tinnitus. *The Journal of Physiology*.

[25] Vogler D. P., Robertson D., Mulders W. H. A. M. (2014). Hyperactivity following unilateral hearing loss in characterized cells in the inferior colliculus. *Neuroscience*.

[26] Kaltenbach J. A., Godfrey D. A., Neumann J. B., McCaslin D. L., Afman C. E., Zhang J. (1998). Changes in spontaneous neural activity in the dorsal cochlear nucleus following exposure to intense sound: relation to threshold shift. *Hearing Research*.

[27] Melamed S. B., Kaltenbach J. A., Church M. W., Burgio D. L., Afmar C. E. (2000). Cisplatin-induced increases in spontaneous neural activity in the dorsal cochlear nucleus and associated outer hair cell loss. *Audiology*.

[28] Kaltenbach J. A., Rachel J. D., Mathog T. A., Zhang J., Falzarano P. R., Lewandowski M. (2002). Cisplatin-induced hyperactivity in the dorsal cochlear nucleus and its relation to outer hair cell loss: relevance to tinnitus. *Journal of Neurophysiology*.

[29] Llinás R., Urbano F. J., Leznik E., Ramírez R. R., van Marle H. J. F. (2005). Rhythmic and dysrhythmic thalamocortical dynamics: GABA systems and the edge effect. *Trends in Neurosciences*.

[30] Houdayer E., Teggi R., Velikova S. (2015). Involvement of cortico-subcortical circuits in normoacousic chronic tinnitus: a source localization EEG study. *Clinical Neurophysiology*.

[31] Weisz N., Muller S., Schlee W., Dohrmann K., Hartmann T., Elbert T. (2007). The neural code of auditory phantom perception. *The Journal of Neuroscience*.

[32] van der Loo E., Gais S., Congedo M. (2009). Tinnitus intensity dependent gamma oscillations of the contralateral auditory cortex. *PLoS One*.

[33] Gopal K. V., Thomas B. P., Nandy R., Mao D., Lu H. (2017). Potential audiological and MRI markers of tinnitus. *Journal of the American Academy of Audiology*.

[34] Chen Y. C., Zhang J., Li X. W. (2014). Aberrant spontaneous brain activity in chronic tinnitus patients revealed by resting-state functional MRI. *NeuroImage: Clinical*.

[35] Llinas R. R., Ribary U., Jeanmonod D., Kronberg E., Mitra P. P. (1999). Thalamocortical dysrhythmia: a neurological and neuropsychiatric syndrome characterized by magnetoencephalography. *Proceedings of the National Academy of Sciences of the United States of America*.

[36] Weisz N., Moratti S., Meinzer M., Dohrmann K., Elbert T. (2005). Tinnitus perception and distress is related to abnormal spontaneous brain activity as measured by magnetoencephalography. *PLoS Medicine*.

[37] Adjamian P., Sereda M., Zobay O., Hall D. A., Palmer A. R. (2012). Neuromagnetic indicators of tinnitus and tinnitus masking in patients with and without hearing loss. *Journal of the Association for Research in Otolaryngology*.

[38] Simmons R., Dambra C., Lobarinas E., Stocking C., Salvi R. (2008). Head, neck, and eye movements that modulate tinnitus. *Seminars in Hearing*.

[39] Pinchoff R. J., Burkard R. F., Salvi R. J., Coad M. L., Lockwood A. H. (1998). Modulation of tinnitus by voluntary jaw movements. *The American Journal of Otology*.

[40] Kraus K. S., Canlon B. (2012). Neuronal connectivity and interactions between the auditory and limbic systems. Effects of noise and tinnitus. *Hearing Research*.

[41] Vanneste S., Plazier M., van der Loo E., van de Heyning P., de Ridder D. (2010). The differences in brain activity between narrow band noise and pure tone tinnitus. *PLoS One*.

[42] Klinge C., Roder B., Buchel C. (2010). Increased amygdala activation to emotional auditory stimuli in the blind. *Brain*.

[43] Cima R. F. F., Maes I. H., Joore M. A. (2012). Specialised treatment based on cognitive behaviour therapy versus usual care for tinnitus: a randomised controlled trial. *The Lancet*.

[44] Beukes E. W., Baguley D. M., Allen P. M., Manchaiah V., Andersson G. (2018). Audiologist-guided internet-based cognitive behavior therapy for adults with tinnitus in the United Kingdom: a randomized controlled trial. *Ear and Hearing*.

[45] Beukes E. W., Allen P. M., Baguley D. M., Manchaiah V., Andersson G. (2018). Long-term efficacy of audiologist-guided internet-based cognitive behavior therapy for tinnitus. *American Journal of Audiology*.

[46] Martinez-Devesa P., Waddell A., Perera R., Theodoulou M. (2020). Cognitive behavioural therapy for tinnitus. *Cochrane Database of Systematic Reviews*.

[47] Londero A., Bonfils P., Lefaucheur J. P. (2018). Transcranial magnetic stimulation and subjective tinnitus. A review of the literature, 2014-2016. *European Annals of Otorhinolaryngology, Head and Neck Diseases*.

[48] Meng Z., Liu S., Zheng Y., Phillips J. S., Cochrane ENT Group (2011). Repetitive transcranial magnetic stimulation for tinnitus. *Cochrane Database of Systematic Reviews*.

[49] Godbehere J., Sandhu J., Evans A. (2019). Treatment of tinnitus using theta burst based repetitive transcranial magnetic stimulation-a single blinded randomized control trial. *Otology & Neurotology*.

[50] Formánek M., Migaľová P., Krulová P. (2018). Combined transcranial magnetic stimulation in the treatment of chronic tinnitus. *Annals of Clinical and Translational Neurology*.

[51] Jacquemin L., Mertens G., van de Heyning P. (2019). An exploratory study on the use of event-related potentials as an objective measure of auditory processing and therapy effect in patients with tinnitus: a transcranial direct current stimulation study. *Otology & Neurotology*.

[52] Lee H. Y. (2019). Adjunctive role of bifrontal transcranial direct current stimulation in distressed patients with severe tinnitus. *Journal of Korean Medical Science*.

[53] Yuan T., Yadollahpour A., Salgado-Ramírez J., Robles-Camarillo D., Ortega-Palacios R. (2018). Transcranial direct current stimulation for the treatment of tinnitus: a review of clinical trials and mechanisms of action. *BMC Neuroscience*.

[54] Hobson J., Chisholm E., El Refaie A. (2012). Sound therapy (masking) in the management of tinnitus in adults. *Cochrane Database of Systematic Reviews*.

[55] Jastreboff P. J., Hazell J. W. P. (2009). A neurophysiological approach to tinnitus: clinical implications. *British Journal of Audiology*.

[56] Phillips J. S., McFerran D. (2010). Tinnitus Retraining Therapy (TRT) for tinnitus. *Cochrane Database of Systematic Reviews*.

[57] Bauer C. A., Berry J. L., Brozoski T. J. (2017). The effect of tinnitus retraining therapy on chronic tinnitus: a controlled trial. *Laryngoscope Investigative Otolaryngology*.

[58] The Tinnitus Retraining Therapy Trial Research Group (2019). Effect of tinnitus retraining therapy vs standard of care on tinnitus-related quality of life a randomized clinical trial. *JAMA Otolaryngology–Head & Neck Surgery*.

[59] NICKEL A. K., Hillecke T., Argstatter H., Bolay H. V. (2005). Outcome research in music therapy: a step on the long road to an evidence-based treatment. *Annals of the New York Academy of Sciences*.

[60] Hanley P. J., Davis P. B. (2008). Treatment of tinnitus with a customized, dynamic acoustic neural stimulus: underlying principles and clinical efficacy. *Trends in Amplification*.

[61] Pantev C., Rudack C., Stein A. (2014). Study protocol: münster tinnitus randomized controlled clinical trial-2013 based on tailor-made notched music training (TMNMT). *BMC Neurology*.

[62] Teismann H., Okamoto H., Pantev C. (2011). Short and intense tailor-made notched music training against tinnitus: the tinnitus frequency matters. *PLoS One*.

[63] Okamoto H., Stracke H., Stoll W., Pantev C. (2010). Listening to tailor-made notched music reduces tinnitus loudness and tinnitus-related auditory cortex activity. *Proceedings of the National Academy of Sciences of the United States of America*.

[64] Stein A., Wunderlich R., Lau P. (2016). Clinical trial on tonal tinnitus with tailor-made notched music training. *BMC Neurology*.

[65] Lee H. Y., Choi M. S., Chang D. S., Cho C. S. (2017). Combined bifrontal transcranial direct current stimulation and tailor-made notched music training in chronic tinnitus. *Journal of Audiology and Otology*.

[66] Kim S. Y., Chang M. Y., Hong M., Yoo S. G., Oh D., Park M. K. (2017). Tinnitus therapy using tailor-made notched music delivered via a smartphone application and Ginko combined treatment: a pilot study. *Auris, Nasus, Larynx*.

[67] Nagaraj M. K., Prabhu P. (2020). Internet/smartphone-based applications for the treatment of tinnitus: a systematic review. *European Archives of Oto-Rhino-Laryngology*.

[68] McNeill C., Távora-Vieira D., Alnafjan F., Searchfield G. D., Welch D. (2012). Tinnitus pitch, masking, and the effectiveness of hearing aids for tinnitus therapy. *International Journal of Audiology*.

[69] Yakunina N., Lee W. H., Ryu Y. J., Nam E. C. (2019). Tinnitus suppression effect of hearing aids in patients with high-frequency hearing loss: a randomized double-blind controlled trial. *Otology & Neurotology*.

[70] Haab L., Lehser C., Corona-Strauss F. I. (2019). Implementation and long-term evaluation of a hearing aid supported tinnitus treatment using notched environmental sounds. *IEEE Journal of Translational Engineering in Health and Medicine*.

[71] Hoare D. J., Edmondson-Jones M., Sereda M., Akeroyd M. A., Hall D. (2014). Amplification with hearing aids for patients with tinnitus and co-existing hearing loss. *Cochrane Database of Systematic Reviews*.

[72] Atkin T., Comai S., Gobbi G. (2018). Drugs for insomnia beyond benzodiazepines: pharmacology, clinical applications, and discovery. *Pharmacological Reviews*.

[73] Bahmad F. M., Venosa A. R., Oliveira C. A. (2006). Benzodiazepines and GABAergics in treating severe disabling tinnitus of predominantly cochlear origin. *The International Tinnitus Journal*.

[74] Han S. S., Nam E. C., Won J. Y. (2012). Clonazepam quiets tinnitus: a randomised crossover study withGinkgo biloba. *Journal of Neurology, Neurosurgery, and Psychiatry*.

[75] Malavasi M., Caovilla H. H., Freitas F. (2002). Clonazepam in the pharmacological treatment of vertigo and tinnitus. *The International Tinnitus Journal*.

[76] Johnson R. M., Brummett R., Schleuning A. (1993). Use of alprazolam for relief of tinnitus. A double-blind study. *Archives of Otolaryngology - Head & Neck Surgery*.

[77] Jalali M. M., Kousha A., Naghavi S. E., Soleimani R., Banan R. (2009). The effects of alprazolam on tinnitus: a cross-over randomized clinical trial. *Medical Science Monitor*.

[78] Puel J. L. (1995). Chemical synaptic transmission in the cochlea. *Progress in Neurobiology*.

[79] Lopez-Gonzalez M. A., Esteban-Ortega F. (2005). Tinnitus dopaminergic pathway. Ear noises treatment by dopamine modulation. *Medical Hypotheses*.

[80] Sziklai I., Szilvassy J., Szilvassy Z. (2011). Tinnitus control by dopamine agonist pramipexole in presbycusis patients: a randomized, placebo-controlled, double-blind study. *Laryngoscope*.

[81] Lopez-Gonzalez M. A., Moliner-Peiro F., Alfaro-Garcia J., Esteban-Ortega F. (2007). Sulpiride plus hydroxyzine decrease tinnitus perception. *Auris Nasus Larynx*.

[82] Lopez-Gonzalez M. A., Santiago A. M., Esteban-Ortega F. (2007). Sulpiride and melatonin decrease tinnitus perception modulating the auditolimbic dopaminergic pathway. *The Journal of Otolaryngology*.

[83] de Azevedo A. A., Langguth B., de Oliveira P. M., Rodrigues Figueiredo R. (2009). Tinnitus treatment with piribedil guided by electrocochleography and acoustic otoemissions. *Otology & Neurotology*.

[84] Luo B., Hu L., Liu C., Guo Y., Wang H. (2016). Activation of 5-HT2A/C receptor reduces glycine receptor-mediated currents in cultured auditory cortical neurons. *Amino Acids*.

[85] Cusack B., Nelson A., Richelson E. (1994). Binding of antidepressants to human brain receptors: focus on newer generation compounds. *Psychopharmacology*.

[86] Sullivan M. D., Sakai C. S., Dobie R. A., Katon W. J. (2016). Treatment of depressed tinnitus patients with nortriptyline. *The Annals of Otology, Rhinology, and Laryngology*.

[87] Sullivan M., Katon W., Russo J., Dobie R., Sakai C. (1993). A randomized trial of nortriptyline for severe chronic tinnitus: effects on depression, disability, and tinnitus symptoms. *JAMA Internal Medicine*.

[88] Podoshin L., Ben-David Y., Fradis M., Malatskey S., Hafner H. (1995). Idiopathic subjective tinnitus treated by amitriptyline hydrochloride/biofeedback. *The International Tinnitus Journal*.

[89] Bayar N., Böke B., Turan E., Belgin E. (2001). Efficacy of amitriptyline in the treatment of subjective tinnitus. *The Journal of Otolaryngology*.

[90] Zoger S., Svedlund J., Holgers K. M. (2006). The effects of sertraline on severe tinnitus suffering--a randomized, double-blind, placebo-controlled study. *Journal of Clinical Psychopharmacology*.

[91] Robinson S. K., Viirre E. S., Bailey K. A., Gerke M. A., Harris J. P., Stein M. B. (2005). Randomized placebo-controlled trial of a selective serotonin reuptake inhibitor in the treatment of nondepressed tinnitus subjects. *Psychosomatic Medicine*.

[92] Mendis D., Johnston M. (2008). An unusual case of prolonged tinnitus following low-dose amitriptyline. *Journal of Psychopharmacology*.

[93] Langguth B., Landgrebe M., Wittmann M., Kleinjung T., Hajak G. (2010). Persistent tinnitus induced by tricyclic antidepressants. *Journal of Psychopharmacology*.

[94] Miller C. W. T. (2016). Development of tinnitus at a low dose of sertraline: clinical course and proposed mechanisms. *Case Reports in Psychiatry*.

[95] Meeus O., De Ridder D., Van de Heyning P. (2011). Administration of the combination clonazepam-Deanxit as treatment for tinnitus. *Otology & Neurotology*.

[96] Brogden R. N., Heel R. C., Speight T. M., Avery G. S. (1981). Trazodone: a review of its pharmacological properties and therapeutic use in depression and anxiety. *Drugs*.

[97] Dib G. C., Kasse C. A., de Andrade T. A., Gurgel Testa J. R., Cruz O. L. M. (2007). Tinnitus treatment with trazodone. *Brazilian Journal of Otorhinolaryngology*.

[98] Hwang S. M., Lim S. H., Oh D. J., Kim S. K., Jung H. H., Im G. J. (2016). Effect of tianeptine on depressed tinnitus patients. *Journal of Audiology and Otology*.

[99] Hudspeth A. (1985). The cellular basis of hearing: the biophysics of hair cells. *Science*.

[100] Moser T., Brandt A., Lysakowski A. (2006). Hair cell ribbon synapses. *Cell and Tissue Research*.

[101] Guitton M. J., Caston J., Ruel J., Johnson R. M., Pujol R., Puel J. L. (2003). Salicylate induces tinnitus through activation of cochlear NMDA receptors. *The Journal of Neuroscience*.

[102] Ruel J., Chabbert C., Nouvian R. (2008). Salicylate enables cochlear arachidonic-acid-sensitive NMDA receptor responses. *The Journal of Neuroscience*.

[103] Sanchez J. T., Ghelani S., Otto-Meyer S. (2015). From development to disease: diverse functions of NMDA-type glutamate receptors in the lower auditory pathway. *Neuroscience*.

[104] Puel J. L., Guitton M. J. (2007). Salicylate-induced tinnitus: molecular mechanisms and modulation by anxiety. *Progress in Brain Research*.

[105] Avan P., Buki B., Petit C. (2013). Auditory distortions: origins and functions. *Physiological Reviews*.

[106] Kennedy H. J., Evans M. G., Crawford A. C., Fettiplace R. (2006). Depolarization of cochlear outer hair cells evokes active hair bundle motion by two mechanisms. *The Journal of Neuroscience*.

[107] Ralli M., Troiani D., Podda M. V. (2014). The effect of the NMDA channel blocker memantine on salicylate-induced tinnitus in rats. *Acta Otorhinolaryngologica Italica*.

[108] Jang C. H., Lee S., Park I. Y., Song A., Moon C., Cho G. W. (2019). Memantine attenuates salicylate-induced tinnitus possibly by reducing NR2B expression in auditory cortex of rat. *Experimental Neurobiology*.

[109] Zheng Y., McNamara E., Stiles L., Darlington C. L., Smith P. F. (2012). Evidence that memantine reduces chronic tinnitus caused by acoustic trauma in rats. *Frontiers in Neurology*.

[110] Lobarinas E., Yang G., Sun W. (2009). Salicylate- and quinine-induced tinnitus and effects of memantine. *Acta Oto-Laryngologica*.

[111] Figueiredo R. R., Langguth B., de Oliveira P. M., de Azevedo A. A. (2008). Tinnitus treatment with memantine. *Otolaryngology and Head and Neck Surgery*.

[112] Olivares D., Deshpande V. K., Shi Y. (2012). N-Methyl D-aspartate (NMDA) receptor antagonists and memantine treatment for Alzheimer’s disease, vascular dementia and Parkinson’s disease. *Current Alzheimer Research*.

[113] Xiong S., Song Y., Liu J. (2019). Neuroprotective effects of MK-801 on auditory cortex in salicylate-induced tinnitus: involvement of neural activity, glutamate and ascorbate. *Hearing Research*.

[114] Criddle M. W., Godfrey D. A., Kaltenbach J. A. (2018). Attenuation of noise-induced hyperactivity in the dorsal cochlear nucleus by pre-treatment with MK-801. *Brain Research*.

[115] Suckfüll M., Althaus M., Ellers-Lenz B. (2011). A randomized, double-blind, placebo-controlled clinical trial to evaluate the efficacy and safety of neramexane in patients with moderate to severe subjective tinnitus. *BMC Ear, Nose and Throat Disorders*.

[116] Azevedo A. A., Figueiredo R. R. (2005). Tinnitus treatment with acamprosate: double-blind study. *Brazilian Journal of Otorhinolaryngology*.

[117] Sharma D. K., Kaur S., Singh J., Kaur I. (2012). Role of acamprosate in sensorineural tinnitus. *Indian Journal of Pharmacology*.

[118] van de Heyning P., Muehlmeier G., Cox T. (2014). Efficacy and safety of AM-101 in the treatment of acute inner ear tinnitus--a double-blind, randomized, placebo-controlled phase II study. *Otology & Neurotology*.

[119] Wu C., Wu X., Yi B. (2018). Changes in GABA and glutamate receptors on auditory cortical excitatory neurons in a rat model of salicylate-induced tinnitus. *American Journal of Translational Research*.

[120] Chambers A. R., Pilati N., Balaram P., Large C. H., Kaczmarek L. K., Polley D. B. (2017). Pharmacological modulation of Kv3.1 mitigates auditory midbrain temporal processing deficits following auditory nerve damage. *Scientific Reports*.

[121] Pilati N., Ison M. J., Barker M. (2012). Mechanisms contributing to central excitability changes during hearing loss. *Proceedings of the National Academy of Sciences*.

[122] Glait L., Fan W., Stillitano G. (2018). Effects of AUT00063, a Kv3.1 channel modulator, on noise-induced hyperactivity in the dorsal cochlear nucleus. *Hearing Research*.

[123] Anderson L. A., Hesse L. L., Pilati N. (2018). Increased spontaneous firing rates in auditory midbrain following noise exposure are specifically abolished by a Kv3 channel modulator. *Hearing Research*.

[124] Hall D. A., Ray J., Watson J. (2019). A balanced randomised placebo controlled blinded phase IIa multi-centre study to investigate the efficacy and safety of AUT00063 versus placebo in subjective tinnitus: the QUIET-1 trial. *Hearing Research*.

[125] Sunwoo W., Jeon Y. J., Bae Y. J., Jang J. H., Koo J. W., Song J. J. (2017). Typewriter tinnitus revisited: the typical symptoms and the initial response to carbamazepine are the most reliable diagnostic clues. *Scientific Reports*.

[126] Dooley D. J., Taylor C. P., Donevan S., Feltner D. (2007). Ca^2+^ channel *α*_2_*δ* ligands: novel modulators of neurotransmission. *Trends in Pharmacological Sciences*.

[127] Bauer C. A., Brozoski T. J. (2006). Effect of gabapentin on the sensation and impact of tinnitus. *Laryngoscope*.

[128] Witsell D. L., Hannley M. T., Stinnet S., Tucci D. L. (2007). Treatment of tinnitus with gabapentin: a pilot study. *Otology & Neurotology*.

[129] Bakhshaee M., Ghasemi M., Azarpazhooh M. (2008). Gabapentin effectiveness on the sensation of subjective idiopathic tinnitus: a pilot study. *European Archives of Oto-Rhino-Laryngology*.

[130] Piccirillo J. F., Finnell J., Vlahiotis A., Chole R. A., Spitznagel E. (2007). Relief of idiopathic subjective tinnitus. *Archives of Otolaryngology – Head & Neck Surgery*.

[131] Dehkordi M. A., Abolbashari S., Taheri R., Einolghozati S. (2019). Efficacy of gabapentin on subjective idiopathic tinnitus: a randomized, double-blind, placebo-controlled trial. *Ear, Nose, & Throat Journal*.

[132] Hoekstra C. E. L., Rynja S. P., van Zanten G. A., Rovers M. M. (2011). Anticonvulsants for tinnitus. *Cochrane Database Syst Rev*.

[133] Koç S., Akyüz S., Somuk B. T. (2016). Paraoxonase activity and oxidative status in patients with tinnitus. *Journal of Audiology & Otology*.

[134] Celik M., Koyuncu I. (2018). A comprehensive study of oxidative stress in tinnitus patients. *Indian Journal of Otolaryngology and Head & Neck Surgery*.

[135] Pawlak-Osińska K., Kaźmierczak H., Marzec M. (2018). Assessment of the state of the natural antioxidant barrier of a body in patients complaining about the presence of tinnitus. *Oxidative Medicine and Cellular Longevity*.

[136] Ekinci A., Kamasak K. (2019). Avaliaçao da atividade da enzima serica prolidase e do estresse oxidativo em pacientes com zumbido. *Brazilian Journal of Otorhinolaryngology*.

[137] Neri S., Signorelli S., Pulvirenti D. (2009). Oxidative stress, nitric oxide, endothelial dysfunction and tinnitus. *Free Radical Research*.

[138] Loiselle A. R., Neustaeter A., de Kleine E., van Dijk P., Jansonius N. M. (2020). Associations between tinnitus and glaucoma suggest a common mechanism: a clinical and population-based study. *Hearing Research*.

[139] Ciorba A., Bianchini C., Pastore A., Mazzoli M. (2013). Pathogenesis of tinnitus: any role for oxidative stress?. *The Journal of International Advanced Otology*.

[140] Poirrier A. L., Pincemail J., Van Den Ackerveken P., Lefebvre P. P., Malgrange B. (2010). Oxidative stress in the cochlea: an update. *Current Medicinal Chemistry*.

[141] Park S. Y., Han J. J., Hwang J. H., Whang E. S., Yeo S. W., Park S. N. (2017). Comparison of tinnitus and psychological aspects between the younger and older adult patients with tinnitus. *Auris Nasus Larynx*.

[142] Gilles A., Ihtijarevic B., Wouters K., van de Heyning P. (2014). Using prophylactic antioxidants to prevent noise-induced hearing damage in young adults: a protocol for a double-blind, randomized controlled trial. *Trials*.

[143] Savastano M., Brescia G., Marioni G. (2007). Antioxidant therapy in idiopathic tinnitus: preliminary outcomes. *Archives of Medical Research*.

[144] Epstein F. H., Vane J. R., Änggård E. E., Botting R. M. (1990). Regulatory functions of the vascular endothelium. *The New England Journal of Medicine*.

[145] De Keulenaer G. W., Andries L. J., Sys S. U., Brutsaert D. L. (1995). Endothelin-mediated positive inotropic effect induced by reactive oxygen species in isolated cardiac muscle. *Circulation Research*.

[146] Lehr H. A., Frei B., Olofsson A. M., Carew T. E., Arfors K. E. (1995). Protection from oxidized LDL-induced leukocyte adhesion to microvascular and macrovascular endothelium in vivo by vitamin C but not by vitamin E. *Circulation*.

[147] Davies K. J., Lin S. W., Pacifici R. E. (1987). Protein damage and degradation by oxygen radicals. IV. Degradation of denatured protein. *The Journal of Biological Chemistry*.

[148] Khan M., Gross J., Haupt H. (2016). A pilot clinical trial of the effects of coenzyme Q10 on chronic tinnitus aurium. *Otolaryngology—Head and Neck Surgery*.

[149] Scasso F., Sprio A. E., Canobbio L. (2017). Dietary supplementation of coenzyme Q10 plus multivitamins to hamper the ROS mediated cisplatin ototoxicity in humans: a pilot study. *Heliyon*.

[150] Niklowitz P., Menke T., Andler W., Okun J. G. (2004). Simultaneous analysis of coenzyme Q10 in plasma, erythrocytes and platelets: comparison of the antioxidant level in blood cells and their environment in healthy children and after oral supplementation in adults. *Clinica Chimica Acta*.

[151] Crane F. L. (2001). Biochemical functions of coenzyme Q10. *Journal of the American College of Nutrition*.

[152] Yeh C. W., Tseng L. H., Yang C. H., Hwang C. F. (2019). Effects of oral zinc supplementation on patients with noise-induced hearing loss associated tinnitus: a clinical trial. *Biomedical Journal*.

[153] Coelho C., Witt S. A., Ji H., Hansen M. R., Gantz B., Tyler R. (2013). Zinc to treat tinnitus in the elderly. *Otology & Neurotology*.

[154] Person O. C., Puga M. E., da Silva E. M., Torloni M. R. (2016). Zinc supplementation for tinnitus. *Cochrane Database of Systematic Reviews*.

[155] Rosenhall U., Skoog B., Muhr P. (2019). Treatment of military acoustic accidents with N-acetyl-L-cysteine (NAC). *International Journal of Audiology*.

[156] El Beaino M., McCaskey M. K., Eter E. (2018). Sulodexide monotherapy in chronic idiopathic subjective tinnitus: a randomized controlled trial. *Otolaryngology–Head and Neck Surgery*.

[157] Incandela L., Cesarone M. R., Belcaro G., De Sanctis M. T. (2002). Treatment of vascular inner ear disease with pentoxifylline: a 4-week, controlled, randomized trial. *Angiology*.

[158] Mahmoudian-Sani M. R., Hashemzadeh-Chaleshtori M., Asadi-Samani M., Luther T. (2017). A review of medicinal plants for the treatment of earache and tinnitus in Iran. *The International Tinnitus Journal*.

[159] Schneider D., Schneider L., Shulman A. (2000). Gingko biloba (Rokan) therapy in tinnitus patients and measurable interactions between tinnitus and vestibular disturbances. *The International Tinnitus Journal*.

[160] Hilton M. P., Zimmermann E. F., Hunt W. T. (2013). Ginkgo biloba for tinnitus. *Cochrane Database of Systematic Reviews*.

[161] Mahmoudian-Sani M. R., Hashemzadeh-Chaleshtori M., Asadi-Samani M., Yang Q. (2017). Ginkgo biloba in the treatment of tinnitus: an updated literature review. *International Tinnitus Journal*.

[162] Procházková K., Šejna I., Skutil J., Hahn A. (2018). Ginkgo biloba extract EGb 761 versus pentoxifylline in chronic tinnitus: a randomized, double-blind clinical trial. *International Journal of Clinical Pharmacy*.

[163] Radunz C. L., Okuyama C. E., Branco-Barreiro F. C. A., Pereira R. M. S., Diniz S. N. (2019). Clinical randomized trial study of hearing aids effectiveness in association with Ginkgo biloba extract (EGb 761) on tinnitus improvement. *Brazilian Journal of Otorhinolaryngology*.

[164] Polanski J. F., Soares A. D., de Mendonca Cruz O. L. (2016). Efeito da terapia com antioxidantes sobre o zumbido em idosos. *Brazilian Journal of Otorhinolaryngology*.

[165] Berkiten G., Yildirim G., Topaloglu I., Ugras H. (2013). Vitamin B12 levels in patients with tinnitus and effectiveness of vitamin B12 treatment on hearing threshold and tinnitus. *B-ENT*.

[166] Shemesh Z., Attias J., Ornan M., Shapira N., Shahar A. (1993). Vitamin B_12_ deficiency in patients with chronic-tinnitus and noise-induced hearing loss. *American Journal of Otolaryngology*.

[167] Singh C., Kawatra R., Gupta J., Awasthi V., Dungana H. (2016). Therapeutic role of vitamin B12 in patients of chronic tinnitus: a pilot study. *Noise & Health*.

[168] Neri G., de Stefano A., Baffa C. (2009). Treatment of central and sensorineural tinnitus with orally administered melatonin and sulodexide: personal experience from a randomized controlled study. *Acta Otorhinolaryngologica Italica*.

[169] Miroddi M., Bruno R., Galletti F. (2015). Clinical pharmacology of melatonin in the treatment of tinnitus: a review. *European Journal of Clinical Pharmacology*.

[170] Hosseinzadeh A., Kamrava S. K., Moore B. C. J. (2019). Molecular aspects of melatonin treatment in tinnitus: a review. *Current Drug Targets*.

[171] Megwalu U. C., Finnell J. E., Piccirillo J. F. (2016). The effects of melatonin on tinnitus and sleep. *Otolaryngology-Head and Neck Surgery*.

[172] Rosenberg S. I., Silverstein H., Rowan P. T., Olds M. J. (1998). Effect of melatonin on tinnitus. *Laryngoscope*.

[173] Hurtuk A., Dome C., Holloman C. H. (2011). Melatonin: can it stop the ringing?. *Annals of Otology, Rhinology & Laryngology*.

[174] Roberts L. E. (2018). Neural plasticity and its initiating conditions in tinnitus. *HNO*.

[175] Shore S. E., Roberts L. E., Langguth B. (2016). Maladaptive plasticity in tinnitus -- triggers, mechanisms and treatment. *Nature Reviews. Neurology*.

[176] Ryan D., Bauer C. A. (2016). Neuroscience of tinnitus. *Neuroimaging Clinics of North America*.

[177] Shore S. E., Wu C. (2019). Mechanisms of noise-induced tinnitus: insights from cellular studies. *Neuron*.

[178] Miyakawa A., Wang W., Cho S. J., Li D., Yang S., Bao S. (2019). Tinnitus correlates with downregulation of cortical glutamate decarboxylase 65 expression but not auditory cortical map reorganization. *Journal of Neuroscience*.

[179] Lopez-Escamez J. A., Bibas T., Cima R. F. F. (2016). Genetics of tinnitus: an emerging area for molecular diagnosis and drug development. *Frontiers in Neuroscience*.

[180] Caspary D. M., Llano D. A. (2017). Auditory thalamic circuits and GABA_A_ receptor function: putative mechanisms in tinnitus pathology. *Hearing Research*.

[181] Norena A. J., Farley B. J. (2013). Tinnitus-related neural activity: theories of generation, propagation, and centralization. *Hearing Research*.

[182] Noreña A. J., Eggermont J., Zeng F. G., Popper A., Fay R. (2012). Stimulating the auditory system to treat tinnitus: from alleviating the symptoms to addressing the causes. *Tinnitus*.

